# Double somatic mutations in *CTNNB1* and *GNA11* in an aldosterone-producing adenoma

**DOI:** 10.3389/fendo.2024.1286297

**Published:** 2024-03-05

**Authors:** Kazutaka Nanba, Amy R. Blinder, Aaron M. Udager, Yuusuke Hirokawa, Takayoshi Miura, Hiroshi Okuno, Koki Moriyoshi, Yuto Yamazaki, Hironobu Sasano, Akihiro Yasoda, Noriko Satoh-Asahara, William E. Rainey, Tetsuya Tagami

**Affiliations:** ^1^ Department of Endocrinology and Metabolism, National Hospital Organization Kyoto Medical Center, Kyoto, Japan; ^2^ Department of Endocrinology, Metabolism, and Hypertension Research, Clinical Research Institute, National Hospital Organization Kyoto Medical Center, Kyoto, Japan; ^3^ Department of Molecular and Integrative Physiology, University of Michigan, Ann Arbor, MI, United States; ^4^ Department of Pathology, University of Michigan, Ann Arbor, MI, United States; ^5^ Michigan Center for Translational Pathology, University of Michigan, Ann Arbor, MI, United States; ^6^ Rogel Cancer Center, University of Michigan, Ann Arbor, MI, United States; ^7^ Department of Radiology, National Hospital Organization Kyoto Medical Center, Kyoto, Japan; ^8^ Department of Urology, National Hospital Organization Kyoto Medical Center, Kyoto, Japan; ^9^ Department of Diagnostic Pathology, National Hospital Organization Kyoto Medical Center, Kyoto, Japan; ^10^ Department of Pathology, Tohoku University Graduate School of Medicine, Sendai, Japan; ^11^ Clinical Research Institute, National Hospital Organization Kyoto Medical Center, Kyoto, Japan; ^12^ Division of Metabolism, Endocrinology, and Diabetes, University of Michigan, Ann Arbor, MI, United States

**Keywords:** primary aldosteronism, aldosterone-producing adenoma, CYP11B2, somatic mutation, CTNNB1, GNA11

## Abstract

Double somatic mutations in *CTNNB1* and *GNA11/Q* have recently been identified in a small subset of aldosterone-producing adenomas (APAs). As a possible pathogenesis of APA due to these mutations, an association with pregnancy, menopause, or puberty has been proposed. However, because of its rarity, characteristics of APA with these mutations have not been well characterized. A 46-year-old Japanese woman presented with hypertension and hypokalemia. She had two pregnancies in the past but had no history of pregnancy-induced hypertension. She had regular menstrual cycle at presentation and was diagnosed as having primary aldosteronism after endocrinologic examinations. Computed tomography revealed a 2 cm right adrenal mass. Adrenal venous sampling demonstrated excess aldosterone production from the right adrenal gland. She underwent right laparoscopic adrenalectomy. The resected right adrenal tumor was histologically diagnosed as adrenocortical adenoma and subsequent immunohistochemistry (IHC) revealed diffuse immunoreactivity of aldosterone synthase (CYP11B2) and visinin like 1, a marker of the zona glomerulosa (ZG), whereas 11β-hydroxylase, a steroidogenic enzyme for cortisol biosynthesis, was mostly negative. CYP11B2 IHC-guided targeted next-generation sequencing identified somatic *CTNNB1* (p.D32Y) and *GNA11* (p.Q209H) mutations. Immunofluorescence staining of the tumor also revealed the presence of activated β-catenin, consistent with features of the normal ZG. The expression patterns of steroidogenic enzymes and related proteins indicated ZG features of the tumor cells. PA was clinically and biochemically cured after surgery. In conclusion, our study indicated that *CTNNB1* and *GNA11*-mutated APA has characteristics of the ZG. The disease could occur in adults with no clear association with pregnancy or menopause.

## Introduction

Aldosterone-producing adenoma (APA) is a major form of primary aldosteronism (PA). In the past decade, there has been significant progress in the determination of genetic causes of APA. The use of next-generation sequencing (NGS) in APA has resulted in the identification of somatic mutations responsible for excess aldosterone production. These affected genes include *KCNJ5* (1), *ATP1A1* (2), *ATP2B3* (2), *CACNA1D* (3, 4), *CACNA1H* (5, 6), and *CLCN2* (7–9). These aldosterone-driver genes encode ion channels or transporters. Mutations in these genes directly or indirectly increase intracellular calcium levels resulting in enhanced tumor cell aldosterone synthase (CYP11B2) expression and inappropriate aldosterone production (10). More recently, somatic mutations in *CADM1* (11) and *SLC30A1* (12) have also been identified as rare genetic causes of APA. An immunohistochemistry (IHC)-based sequencing approach that targets CYP11B2-expressing regions using formalin-fixed, paraffin-embedded (FFPE) tissue has enabled detection of these somatic mutations in the vast majority of APAs (13–16).

As in other adrenocortical tumors such as adrenocortical carcinoma and cortisol-producing adenoma, somatic activating mutations in exon 3 of the *CTNNB1* gene, that encodes β-catenin, have also been identified in 2-5% of APA (17–19). A recent study reported double somatic mutations of *GNA11* or *GNAQ* in *CTNNB1*-mutated APAs (20). As a possible pathogenesis of APA harboring these double mutations, an association with pregnancy, menopause, or puberty has been proposed based on the disease onset and increased tumor expression of luteinizing hormone/choriogonadotropin receptor (LHCGR) (20). However, due to its rare incidence, characteristics of APA with these double mutations have not been well characterized. Herein, we report the detailed clinical course of a Japanese woman with APA harboring somatic *CTNNB1* and *GNA11* mutations. Notably, the present case had no history of pregnancy-associated hypertension or irregular menstrual cycles at presentation.

## Materials and methods

### Immunohistochemistry

IHC was performed on 10% FFPE tissue sections as described previously (21). The following primary antibodies were used: CYP11B2 (MilliporeSigma, MABS1251; diluted 1:1250; RRID, AB_2783793), 17α-hydroxylase/17, 20 lyase (CYP17A1) (LSBio, LS-B14227; diluted 1:2000; RRID, AB_2857939), 11β-hydroxylase (CYP11B1) (clone 80-7-3; kindly provided by Dr. Celso Gomez-Sanchez; diluted 1:50; RRID, AB_2650563), and visinin like 1 (VSNL1) (MilliporeSigma, MABN762; diluted 1:1000; RRID, AB_2832208).

### Immunofluorescence staining

Immunofluorescence (IF) was performed on FFPE sections of 5 μm thickness. After deparaffinization, the slides were boiled for 15 minutes in pH 6, citrate-based buffer (Vector Laboratories) for epitope retrieval followed by 10% normal goat serum (Abcam) blocking for 1 hour. The primary antibodies to β-catenin (Cell Signaling Technology, 9562; diluted 1:100; RRID, AB_331149) and KCNJ5 (G protein-activated inward rectifier potassium channel 4) (from Dr. Celso Gomez-Sanchez; clone 36-33-5; diluted 1:1000; RRID, AB_3086774) (22) were incubated overnight at 4˚C. The fluorescent-conjugated secondary antibodies (Jackson ImmunoResearch, 111-545-144; diluted 1:100; RRID, AB_2338052 and Thermo Fisher Scientific, A-11032; diluted 1:100; RRID, AB_2534091) were then incubated for 1 hour at room temperature followed by autofluorescence quenching with TrueBlack® Lipofuscin Autofluorescence Quencher (Biotium) for 30 seconds. Finally, coverslips were mounted with 4’,6-diamidino-2-phenylindole (DAPI).

### DNA and RNA isolation

Genomic DNA (gDNA) and RNA from APA and adjacent normal adrenal tissue were isolated separately from serial FFPE tissue sections using the AllPrep DNA/RNA FFPE kit (QIAGEN) as described previously (23). gDNA and RNA were used for targeted NGS and quantitative real-time RT-PCR (qPCR), respectively.

### Targeted NGS

Ion Torrent™-based targeted NGS (Thermo Fisher Scientific) was used for sequencing analysis. The custom Ion AmpliSeq™ panel for targeted NGS included the full coding regions of following genes: *KCNJ5*, *ATP1A1*, *ATP2B3*, *CACNA1D*, *CACNA1H*, *CLCN2*, *CADM1*, *SLC30A1*, *CTNNB1*, *GNAS*, and *GNA11*. The methods for targeted NGS, including library preparation, sequencing, and variant calling, were performed as described previously (23).

### Quantitative real-time RT-PCR

RNA was reverse transcribed using the high-capacity complementary DNA (cDNA) archive kit (Life Technologies). qPCR was performed using the StepOnePlus™ Real-Time PCR systems (Applied Biosystems) (23). The primer-probe sets for *CYP11B2* were designed in house and manufactured by IDT DNA (24). The following primer-probe sets were purchased from Thermo Fisher Scientific: *LHCGR* (Hs00174885_m1), *GNRHR* (gonadotropin-releasing hormone receptor) (Hs00171248_m1), and *ACTB* (β-actin) (Hs01060665_g1). *ACTB* transcript was used as an internal control for quantitative normalization. The delta-delta threshold cycle method was used to calculate fold changes in mRNA expression over adjacent normal adrenal.

This study was approved by the institutional review boards at the National Hospital Organization Kyoto Medical Center (20–038) and the University of Michigan (HUM00083056). The patient provided written consent for the use of specimen in this study and publication of this article.

## Results

### Case presentation

A 46-year-old Japanese woman was referred to us for the investigation of PA. She had been hypertensive at least for 4 months (her blood pressure was 216/105 mmHg at initial visit of the referring hospital). She had two pregnancies at the ages of 22 and 23 but had no history of pregnancy-associated hypertension or other complications according to her Maternal and Child Handbooks (25). Although she had menopause-like symptoms such as headaches, sweating, and fatigue, her menstrual cycle was regular at the time of presentation. She had urolithiasis at the age of 40. Computed tomography (CT) for the evaluation of urolithiasis detected a right adrenal tumor. However, no further investigation was performed at that time. She had no family history of endocrine disorders.

**Figure 1:**
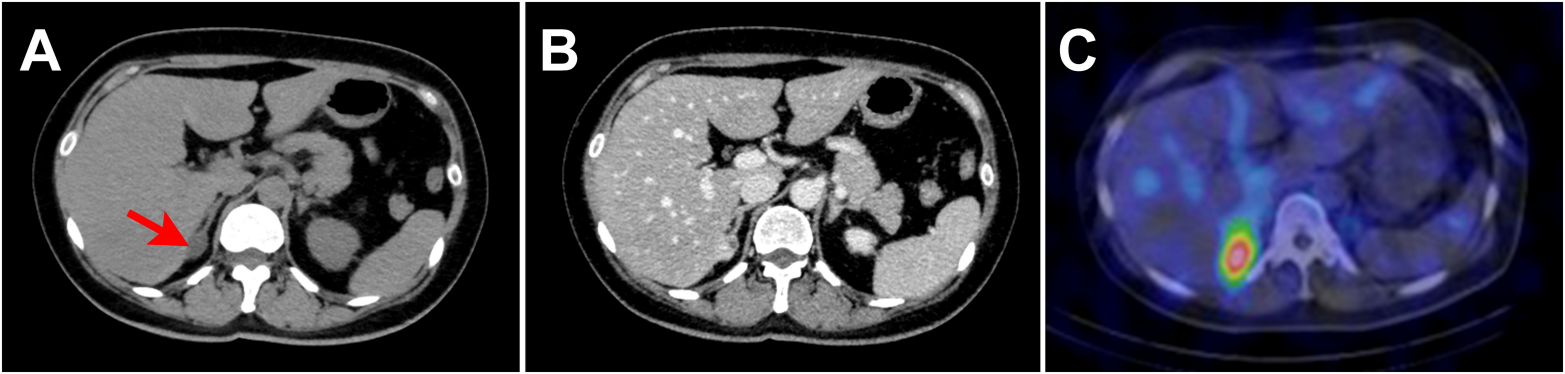
Imaging findings. **(A, B)**. Computed tomography (CT) revealed a 2 cm right adrenal mass (red arrow in **(A)**). The mean Hounsfield unit of the adrenal tumor on unenhanced CT was 14.0. **(A)**. Unenhanced CT. **(B)**. Contrast enhanced CT. **(C)**. NP-59 scintigraphy with dexamethasone suppression showed increased tracer uptake in the right adrenal lesion.

**Figure 2:**
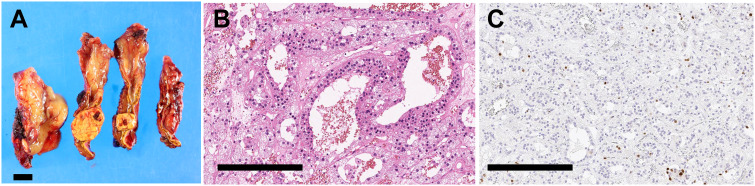
Histopathology of resected adrenal tumor. **(A).** Cut surfaces of resected adrenal tissue showing a yellow nodule with a diameter of 2.0 cm. Scale bar, 1 cm. **(B, C)**. High magnification photomicrographs of adrenal tumor. Scale bars, 300 µm. **(B)**. Hematoxylin and eosin staining. **(C)** Ki-67 staining.

Laboratory testing showed hypokalemia and elevated plasma aldosterone concentration with suppressed renin (**Table 1**). She was diagnosed as having PA based on the results of captopril challenge test (**Table 1**) (26). Concomitant cortisol excess was not documented (**Table 1**). Adrenal CT revealed a 2 cm right adrenal mass (**Figures 1A, B**). Left adrenal was intact by imaging. Adrenal venous sampling indicated excess aldosterone production from the right adrenal gland (**Table 2**). ^131^I-6β-iodomethyl-19-norcholesterol (NP-59) scintigraphy with dexamethasone suppression further demonstrated increased tracer uptake in the right adrenal lesion (**Figure 1C**). She underwent right laparoscopic adrenalectomy. The resected tumor was histologically diagnosed as adrenocortical adenoma according to the criteria of Weiss (27) and also harboring the foci of pseudoglandular formations (**Figures 2A, B**). Notably, Ki-67 labeling index was high (6% at hotspots) (**Figure 2C**). After surgery, her blood pressure and serum potassium were both normalized. Based on the primary aldosteronism surgical outcome (PASO) study criteria (28), PA was clinically and biochemically cured after surgery (**Table 3**). No tumor recurrence was observed by imaging study performed at 2 years after surgery.

**Table 1:** Laboratory results of endocrine testing.

	Values
Baseline characteristics	
Serum creatinine (mg/dL)	0.61
Serum potassium (mEq/L)	2.9
PAC (ng/dL)	67.8
PRA (ng/mL/h)	0.3
ARR	226.0
Captopril challenge test^a^	
Baseline PAC (ng/dL)	101.2
Baseline PRA (ng/mL/h)	0.6
Baseline ARR	168.7
60 min PAC (ng/dL)	63.9
60 min PRA (ng/mL/h)	0.4
60 min ARR	159.8
90 min PAC (ng/dL)	52.4
90 min PRA (ng/mL/h)	0.5
90 min ARR	104.8
ACTH/cortisol circadian rhythm	
8:00 ACTH (pg/mL)	37.3
8:00 serum cortisol (μg/dL)	8.6
23:00 ACTH (pg/mL)	8.2
23:00 serum cortisol (μg/dL)	1.1
1 mg dexamethasone suppression test^b^	
ACTH (pg/mL)	<1.5
Serum cortisol (μg/dL)	0.9
PAC (ng/dL)	106.1

^a^, ARR ≥ 20 at 60 or 90 minutes after 50 mg of captopril administration was considered as a positive result (26). ^b^, A cut-off cortisol level of ≥ 1.8 μg/dL was used to assess the presence of autonomous cortisol co-secretion (26). PAC, plasma aldosterone concentration; PRA, plasma renin activity; ARR, aldosterone-to-renin ratio; ACTH, adrenocorticotropic hormone.

**Table 2:** Results of adrenal venous sampling.

	Values
Right AV PAC (ng/dL)	1606.4
Right AV cortisol (µg/dL)	257
Left AV PAC (ng/dL)	257.3
Left AV cortisol (µg/dL)	498
IVC PAC (ng/dL)	115.1
IVC cortisol (µg/dL)	21.7
Selectivity index (right)	11.8
Selectivity index (left)	22.9
A/C (right AV)	6.25
A/C (left AV)	0.52
A/C (IVC)	5.3
Lateralized ratio	12.0
Contralateral ratio	0.10

Adrenal venous sampling was performed under cosyntropin stimulation. Selectivity index ≥ 5.0 was used as a cut-off for successful catheterization (26). Lateralized index > 4.0 was used as a cut-off for lateralized disease (26). AV, adrenal vein; PAC, plasma aldosterone concentration; IVC, inferior vena cava; A/C, aldosterone to cortisol ratio.

**Table 3:** Post-operative clinical course.

	Post-operative data						
	1 month	3 months	6 months	12 months	18 months	24 months	30 months
Blood pressure (mmHg)	104/68	128/77	104/42	127/80	108/66	98/63	106/77
Serum creatinine (mg/dL)	0.66	0.68	0.73	0.66	0.72	0.66	0.67
Serum potassium (mEq/L)	4.4	4.2	4.9	4.0	4.6	4.3	4.3
PAC (ng/dL)	12.8	18.3	28.3	20.3	16.2^a^	13.2	7.9
PRA (ng/mL/h)	0.9	1.6	2.4	1.3	1.3	0.7	0.7
ARR	14.2	11.4	11.8	15.6	12.5	18.9	11.3

^a^, Assay kit for PAC measurement (chemiluminescent enzyme immunoassay) was changed from the Accuraseed Aldosterone kit (FUJIFILM Wako Pure Chemical Corp, Japan) to the Accuraseed Aldosterone·S kit (FUJIFILM Wako Pure Chemical Corp, Japan) from this point. PAC, plasma aldosterone concentration; PRA, plasma renin activity; ARR, aldosterone-to-renin ratio.

### Histopathologic and genetic characteristics of the resected tumor

**Figure 3:**
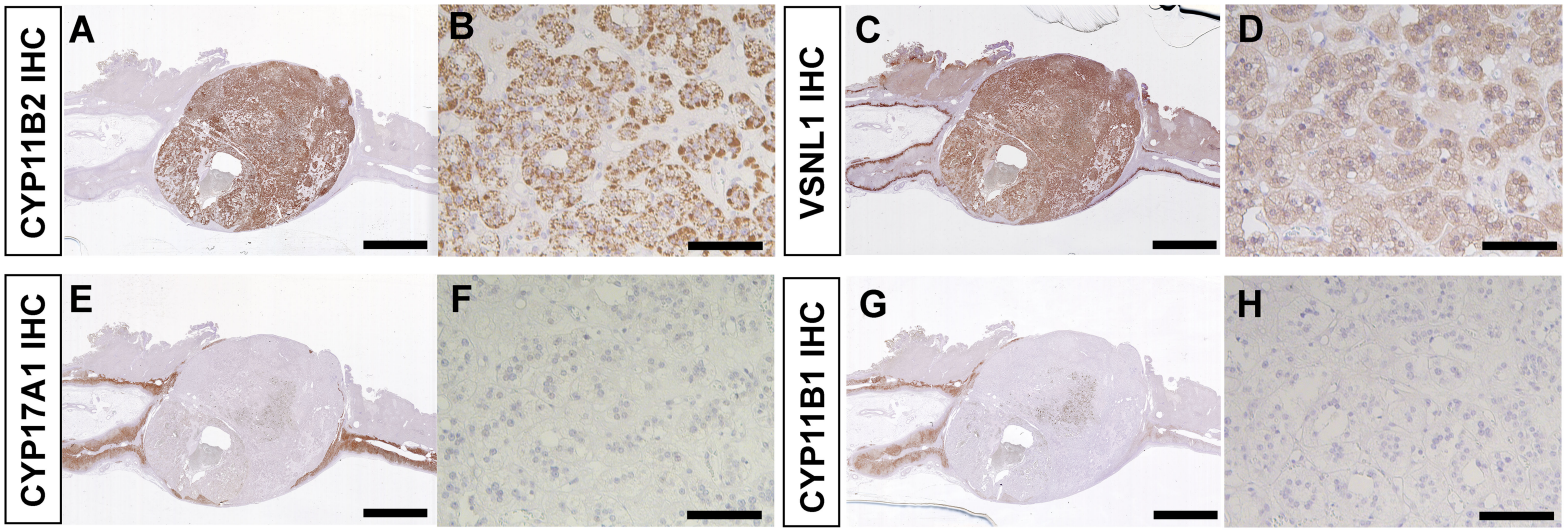
Immunohistochemistry of resected adrenal tumor. **(A, B)**. CYP11B2 IHC. **(C, D)**. VSNL1 IHC. **(E**, **F)**. CYP17A1 IHC. **(G, H)**. CYP11B1 IHC. **(A, C, E, G)**. Scanned images of stained slides. Scale bars, 5 mm. **(B, D, F, H)**. High magnification photomicrographs of adrenal tumor. Scale bars, 100 µm.

IHC revealed diffuse immunoreactivity of CYP11B2 in tumor cells suggestive of neoplastic production of aldosterone (**Figures 3A, B**). VSNL1, a marker for the normal zona glomerulosa (ZG) (29), was also abundant in the tumor (**Figures 3C, D**). Consistent with normal suppression of cortisol after 1 mg dexamethasone suppression test, immunoreactivity of CYP17A1 and CYP11B1, both required for cortisol biosynthesis, was markedly low (**Figures 3E–H**). The adjacent adrenal tissue demonstrated paradoxical hyperplasia of the ZG, a hyperplastic ZG with negative CYP11B2 immunoreactivity, and aldosterone-producing micronodules (30). There were no atrophic changes in the zona fasciculata (ZF) or zona reticularis (ZR) of the adjacent adrenal tissue (**Supplementary Figure 1A**). In the ZR, normal dehydroepiandrosterone sulfotransferase (DHEA-ST) immunoreactivity was observed (**Supplementary Figure 1B**).

Targeted NGS identified double somatic *CTNNB1* (p.D32Y) and *GNA11* (p.Q209H) mutations with similar variant allele frequencies (**Table 4**). Using our method, these mutations were not detected in adjacent adrenal gDNA, suggesting their somatic origin. qPCR revealed high tumor expression of *CYP11B2* mRNA (599-fold over adjacent normal adrenal), confirming accurate sample collection. In agreement with previous studies (20, 31), *LHCGR* and *GNRHR* mRNA levels were also elevated within the tumor compared with those in adjacent normal adrenal (148-fold and 56-fold, respectively).

**Table 4:** Results of targeted NGS.

Gene	Exon	Nucleotide change	Amino acid change	FDP	VAF (%)	Reference sequence
*CTNNB1*	3	c.G94T	p.D32Y	1997	29.5	NM_001904
*GNA11*	5	c.G627C	p.Q209H	2000	29.7	NM_002067

FDP, flow-corrected read depth; VAF, variant allele frequency.

**Figure 4:**
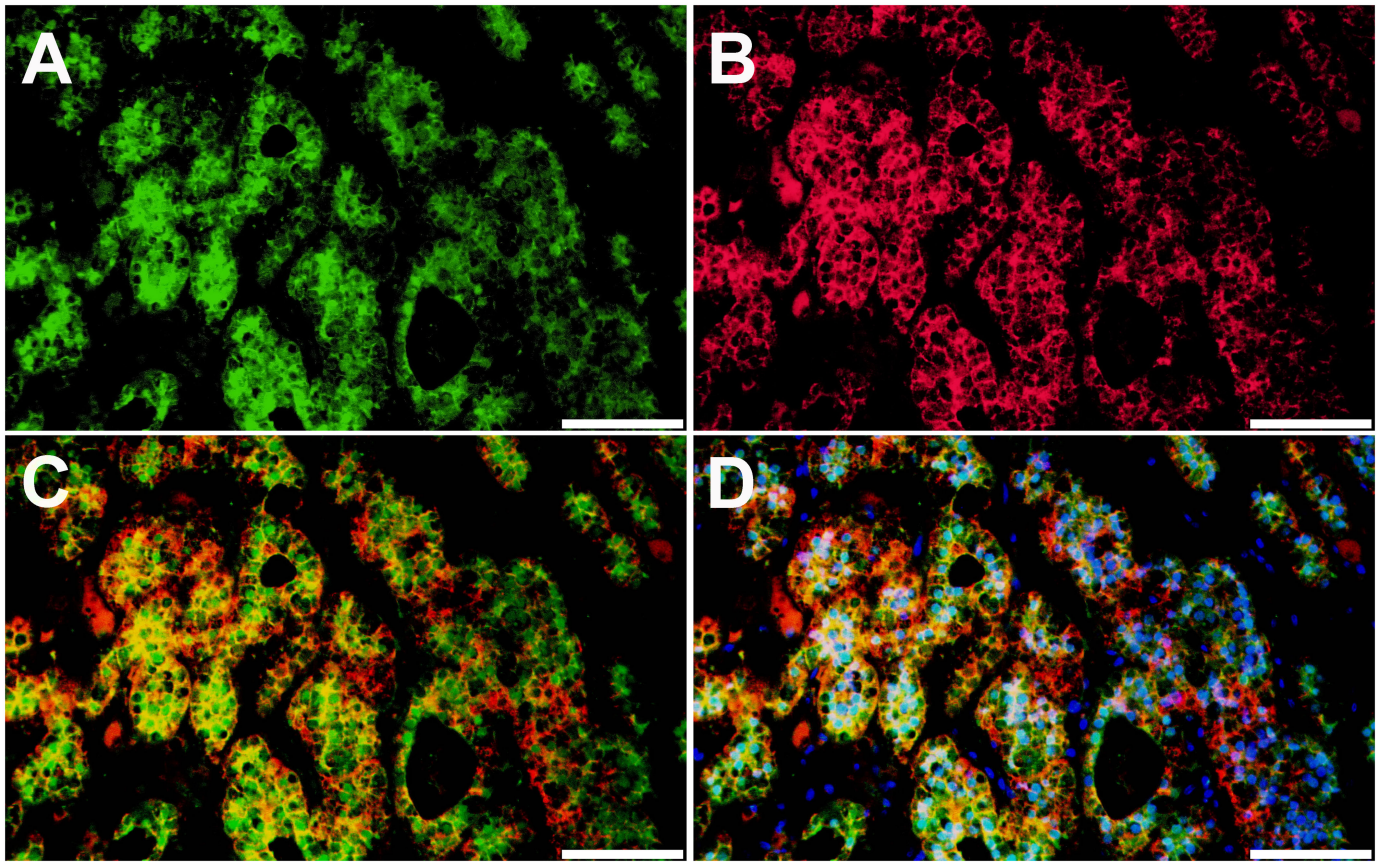
Localization of β-catenin protein in aldosterone-producing adenoma harboring somatic *CTNNB1* and *GNA11* mutations. β-catenin protein expression patterns in aldosterone-producing adenoma were assessed by immunofluorescence (IF) staining. **(A)**. IF for β-catenin (β-catenin, green). **(B)**. IF for KCNJ5 (KCNJ5, red). **(C)**. IF for β-catenin and KCNJ5. **(D)**. IF for β-catenin and KCNJ5 with DAPI (DAPI, blue). Scale bars, 100 µm.

We further tested β-catenin protein localization using IF staining to assess Wnt/β-catenin activation status (**Figures 4A–D**). In IF staining, KCNJ5 was used as a plasma membrane marker. A subset of tumor cells revealed nuclear and/or cytoplasmic immunoreactivity of β-catenin, suggesting activated status, which is seen in the ZG of normal adrenal glands (32).

## Discussion

The Wnt/β-catenin signaling pathway plays an important role in adrenocortical development, homeostasis, and regeneration (33). In the non-pathologic human adrenal cortex, activated β-catenin (nuclear and/or cytoplasmic expression) is restricted to the ZG, where physiologic aldosterone biosynthesis occurs. In contrast, non-activated β-catenin (cell membrane expression) is predominant in the ZF (32). Aberrant Wnt/β-catenin signaling was reported to lead to various adrenal disorders and dysregulated steroidogenesis (33). Although the prevalence of somatic *CTNNB1* mutation is relatively low in APA, activated β-catenin, i.e., nuclear and/or cytoplasmic localization of β-catenin, was reported in the majority of APA (34). A recent study investigating intra-tumor heterogeneity in APA demonstrated that β-catenin was activated mainly in CYP11B2-expressing regions of the tumor (16). The adrenal tumor from the present case showed diffuse CYP11B2 immunoreactivity. Like ZG cells, a subset of tumor cells demonstrated rosette-like structure and activated β-catenin (**Figure 4**). The intense tumor expression of VSNL1, one of the ZG markers, also supports a ZG identity of the tumor (**Figures 3C, D**).

Zhou et al. (20) recently demonstrated the coexistence of gain-of-function mutations in *GNA11* or its close homolog, *GNAQ*, in 16 of 27 *CTNNB1*-mutated APAs (59%). The *GNA11* and *GNAQ* genes encode G-protein subunit alpha 11 (G11) and G-protein subunit alpha q (Gq), respectively. Gq/11 act as important modulators of angiotensin II receptor activation, which is one of the main physiologic regulators of aldosterone production in ZG cells (35). The mutations in *GNA11/Q* in APA have always been detected in the highly conserved p.Q209 residue that is crucial for GTPase activation. These mutations inhibit GTPase activity, resulting in constitutive activation of downstream signaling and enhanced aldosterone production (20). High tumor expression of LHCGR and GNRHR in APAs with *CTNNB1* (and *GNA11/Q*) mutations has been a rationale for the link between the disease onset and pregnancy, menopause, or puberty (20, 31). In Zhou’s study above, double mutations of *CTNNB1* and *GNA11/Q* were more often seen in women than men (15 vs. 1) and the disease onset of 12 out of 16 cases (75%) was associated with pregnancy, menopause, or puberty (20). Our present case also showed elevated expression of *LHCGR* and *GNRHR* mRNA in the tumor compared with that in adjacent adrenal. However, the pathophysiologic role of high tumor expression of *LHCGR* and *GNRHR* mRNA in our case is unclear since her disease onset was not directly associated with pregnancy or menopause. Of particular note, one of the 16 cases in Zhou’s study had the same combination of mutations as our case (*CTNNB1* p.D32Y and *GNA11* p.Q209H) and the patient had no history of hypertension during her past 10 pregnancies (20).

Previous studies also reported aberrant expression of G protein-coupled receptors, including *LHCGR*, *GNRHR*, 5-hydroxytryptamine (serotonin) receptor 4 *(HTR4)*, and melanocortin 2 receptor *(MC2R)* in APAs (36, 37). In addition, some of patients with PA were reported to show enhanced aldosterone production in response to luteinizing hormone (LH), human chorionic gonadotropin (hCG), or gonadotropin-releasing hormone (GnRH) (38–42). Gagnon et al. (41) investigated genetic characteristics of GnRH/LH-responsive PA, including APA, bilateral macronodular adrenal hyperplasia, and other rarer forms. In their cohort, 17 patients with APA underwent *in vivo* GnRH and/or LH tests; 6, 10, and 1 had both, only GnRH, and only LH tests, respectively. Among 16 APAs tested for GnRH, 6 and 3 APAs showed positive and partial response, respectively. Positive response to LH was observed in 5 out of 7 APAs tested. Sequencing analysis of 15 APAs that had *in vivo* GnRH and/or LH tests revealed 3 *KCNJ5* (1 tested for GnRH and LH, no response; 1 tested for GnRH, partial response; 1 tested for LH, positive response), 1 *ATP1A1* (tested for GnRH, no response), and 1 *CACNA1D* mutations (tested for GnRH, no response). Of particular interest, there were no *CTNNB1*-mutated APAs in their cohort (41). Another study by Kishimoto et al. (40) demonstrated that *GNRHR* and *LHCGR* mRNA levels were higher and the response to GnRH was greater in APAs with no known mutations (mutation hotspots of *KCNJ5, ATP1A1, ATP2B3, CACNA1D*, and *CTNNB1* genes were screened) (n=9) compared with those with *KCNJ5* hotspot mutations (n=13). Genetic causes of GnRH/LH-responsive APAs appear to be heterogeneous and largely unknown. Further dedicated studies are needed.

Because of its rare incidence, clinical characteristics of the patients with APA harboring double *CTNNB1* and *GNA11/Q* mutations are not well characterized. Our case had typical clinical characteristics of PA with no excess cortisol co-secretion. Although the histologic findings were compatible with adrenocortical adenoma according to the criteria of Weiss (27), the tumor cells had unusually high Ki-67 labeling index for an adenoma (43). The present case was therefore closely followed up after surgery. Post-operative clinical course was indeed excellent with achievement of clinical and biochemical cure and no tumor recurrence was observed. Our present case also indicates that the occurrence of APA with double *CTNNB1* and *GNA11* somatic mutations is not always associated with pregnancy or menopause. In conclusion, we present a case of APA with double somatic mutations in *CTNNB1* and *GNA11*. Detailed clinical and histologic examination will provide useful information for better characterization of patients with PA caused by these rare mutations.

## Data availability statement

The datasets presented in this article are not readily available because of ethical and privacy restrictions. Requests to access the datasets should be directed to the corresponding author.

## Ethics statement

The studies involving humans were approved by National Hospital Organization Kyoto Medical Center and the University of Michigan. The studies were conducted in accordance with the local legislation and institutional requirements. The participants provided their written informed consent to participate in this study. Written informed consent was obtained from the individual(s) for the publication of any potentially identifiable images or data included in this article.

## Author contributions

All authors made individual contributions to authorship. KN and WR conceived the idea of molecular analysis. KN drafted the manuscript. AB and AU performed molecular analysis. KN, YH, TM, and HO were involved in the care of the patient. KM, YY, and HS were involved in histologic diagnosis. AY, NS-A, and TT provided input for the case and manuscript. All authors contributed to the article and approved the submitted version.
