# Supplementary material for: Double somatic mutations in CTNNB1 and GNA11 in an aldosterone-producing adenoma
Source: Front Endocrinol (Lausanne). 2024 Mar 5;15:1286297. doi: 10.3389/fendo.2024.1286297 (PMC10948454; doi:10.3389/fendo.2024.1286297)
Supplement: Supplementary file 1 [file Image_1.pdf]

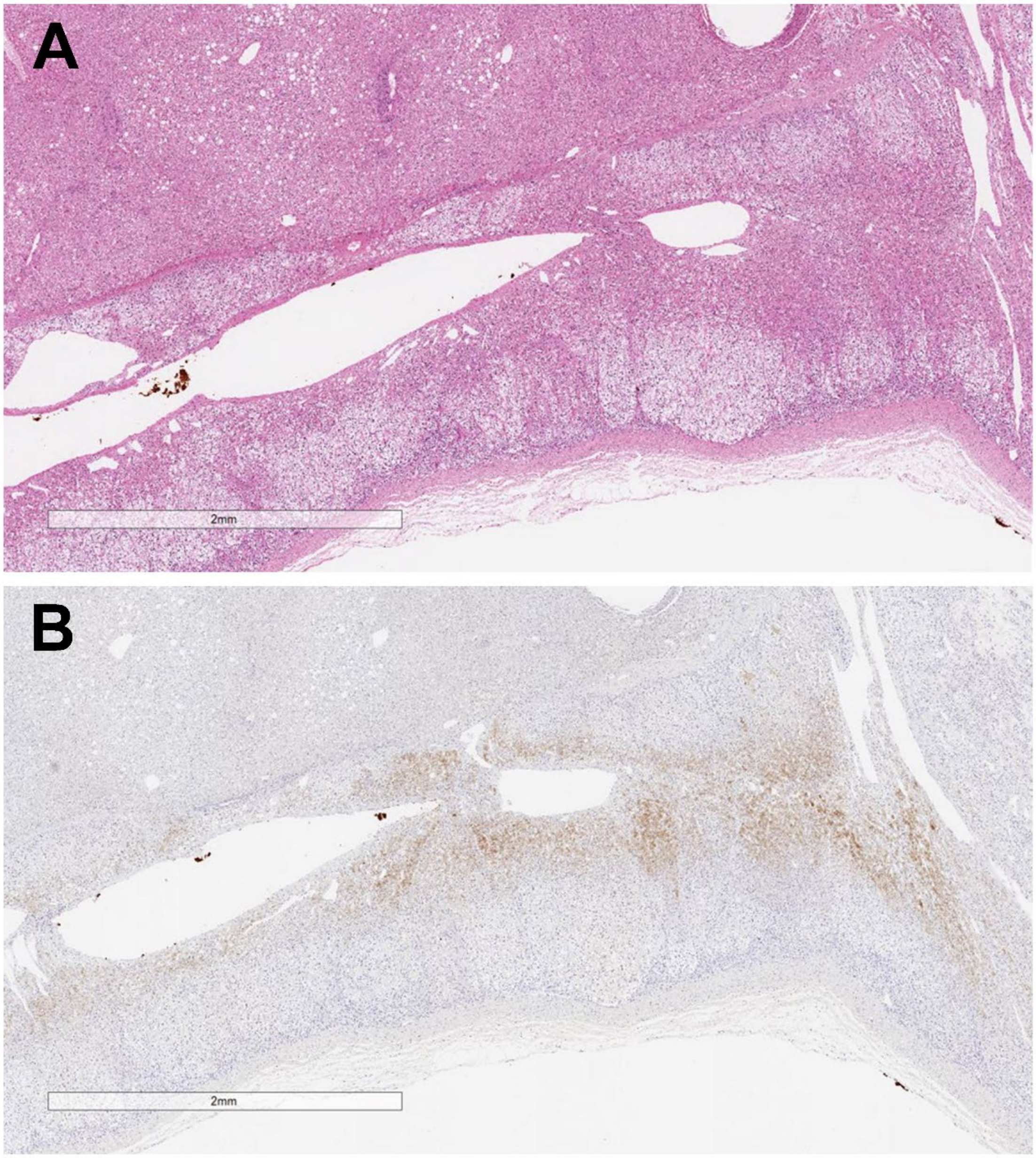

Supplementary Figure 1. Histologic findings of the adjacent adrenal tissue.

A, Hematoxylin and eosin staining. **B,** DHEA-ST immunohistochemistry.
